# Developmental Changes in the Role of Different Metalinguistic Awareness Skills in Chinese Reading Acquisition from Preschool to Third Grade

**DOI:** 10.1371/journal.pone.0096240

**Published:** 2014-05-08

**Authors:** Tong-Qi Wei, Hong-Yan Bi, Bao-Guo Chen, Ying Liu, Xu-Chu Weng, Taeko N. Wydell

**Affiliations:** 1 Key Laboratory of Behavioral Science, Institute of Psychology, Chinese Academy of Sciences, Beijing, China; 2 Beijing Key Lab of Applied Experimental Psychology, School of Psychology, Beijing Normal University, Beijing, China; 3 School of Health Care Management and Education, China Capital Medical University, Beijing, China; 4 Hangzhou Normal University, Central of Cognition & Brain Disorders, Hangzhou, Zhejiang, China; 5 Centre for Cognition and Neuroimaging, Department of Psychology, School of Social Sciences, Brunel University, Middlesex, United Kingdom; Utrecht University, Netherlands

## Abstract

The present study investigated the relationship between Chinese reading skills and metalinguistic awareness skills such as phonological, morphological, and orthographic awareness for 101 Preschool, 94 Grade-1, 98 Grade-2, and 98 Grade-3 children from two primary schools in Mainland China. The aim of the study was to examine how each of these metalinguistic awareness skills would exert their influence on the success of reading in Chinese with age. The results showed that all three metalinguistic awareness skills significantly predicted reading success. It further revealed that orthographic awareness played a dominant role in the early stages of reading acquisition, and its influence decreased with age, while the opposite was true for the contribution of morphological awareness. The results were in stark contrast with studies in English, where phonological awareness is typically shown as the single most potent metalinguistic awareness factor in literacy acquisition. In order to account for the current data, a three-stage model of reading acquisition in Chinese is discussed.

## Introduction

Children typically develop literacy skills in literate societies with appropriate education. However, there are some children who fail to acquire reading proficiency, who tend to have associated behavioral, academic, and psychological difficulties [1]. Many distinct cognitive psychological factors play key roles in reading development, such as, metalinguistic awareness [2], executive function [3], grammatical skills [4], naming speed [5], [6] and so on. The relationships between these factors and literacy acquisition vary across different languages. For example, non-alphabetic languages such as Chinese with a deep orthography show differences in the way these cognitive psychological processes interact with each other in reading and in the acquisition of literacy when compared with alphabetic languages such as English which is also considered to have a deep orthography [7]–[9].

### Phonological Awareness and Reading Development in Chinese

Extensive research into the cognitive processes involved in reading has shown that in alphabetic languages phonological awareness plays a dominant role (e.g., [10], [11]). A similar relationship between phonological awareness and reading achievement has also been reported by researchers who are interested in the acquisition of reading in Chinese in Hong Kong [12], Taiwan [13], and Mainland China [14]. Ziegler et al. [15] concluded in a cross-linguistic study that the impact of phonological awareness was modulated by the orthographic depth, i.e., the impact of phonological awareness was stronger in less transparent orthographies (see [16] for a similar suggestion). Although the study did not include non-alphabetic scripts, Ziegler et al. speculated that this conclusion could be applied to Chinese. Thus they further suggested that similar research needs to be conducted in Chinese, given that the characteristics of Chinese orthography differ significantly from those of English. While recognizing the general importance of phonological awareness for reading acquisition/development in Chinese and English, other researchers found that phonological awareness skills did not always have a dominant contribution to reading development in Chinese. Ho, Chan, Lee, Tsang and Luan [17] tested 147 primary school children with developmental dyslexia in Hong Kong on a number of literacy and cognitive measures. They found that compared to typically developing children, these dyslexic children were significantly poorer in rapid naming and orthographic processing skills, and only a small proportion of these Chinese dyslexic children had a phonological deficit. Ho et al. further asserted that “orthographic-related difficulties may be the crux of the problem in Chinese developmental dyslexia” (p. 70). This conclusion was further supported by the event-related potential experiments conducted by Wang, Bi, Gao, and Wydell [18] which linked orthographic difficulties arising from an abnormality in the visual magnocellular system in the Chinese individuals with developmental dyslexia. A cross-linguistic study conducted by McBride-Chang et al. [19] found that word recognition skills were significantly related to morphological awareness rather than phonological awareness in Chinese children in Beijing and Hong Kong, whereas phonological awareness was the only significant predictor of reading in English-speaking children in the United States. Given these contradictory conclusions, it is necessary to consider further the influence of morphological and orthographic awareness skills in the acquisition of literacy in Chinese.

### Morphological Awareness and Reading Development in Chinese

Morphological awareness is defined as children's conscious awareness of the morphemic structure of words and the meaning of morphemes, which are the smallest meaningful linguistic units [20], [21]. The main feature of Chinese morphology is monosyllabism. This means that morphological changes in Chinese are relatively rarer compared to the pervasive morphological changes in Indo-European languages [22]. Most Chinese words are formed by combining morphemes directly rather than via derivations and inflections, and their meaning can be inferred from the combining of morphemes in regular and predictable ways [23].

An increasing body of research has shown converging evidence for the importance of morphological awareness in reading Chinese. McBride-Chang, Shu, Zhou, Wat and Wagner [24] found that after controlling the variables such as age, phonological awareness, speeded naming, speed of processing and vocabulary, morphological awareness was a potent predictor for Chinese character recognition. Further, Shu, McBride-Chang, Wu and Liu [25] found that in comparing children with developmental dyslexia and typically developing children, morphological awareness not only acts as the most discriminating variable, but also consistently the strongest predictor of character recognition, writing to character dictation and comprehension. The importance of morphological awareness was also acknowledged by Wu, Anderson, Li, et al. [26], who reported that morphological awareness instruction improved literacy, and that the relationship between them was bidirectional in Grade-3 children (aged eight) in Mainland China. All these findings emphasize morphological awareness as a unique, important metalinguistic awareness skill in the literacy acquisition in Chinese.

### Orthographic Awareness and Reading Development in Chinese

Orthographic awareness refers to the reading processes involved in forming, storing, and accessing the orthographic representations of a language [27]. Chinese orthographic awareness is defined as children's understanding of the orthographic rules for Chinese characters. More than 80% of Chinese characters are ideo-phonetic characters, which usually consist of a phonetic radical and a semantic radical. The phonetic radical provides information as to the character's pronunciation. Without considering the tone of the characters, the pronunciations of more than half of ideo-phonetic characters are congruent with their phonetic radical. Semantic radicals provide approximate information about a character's meaning, or at least the semantic category of the characters they belong to [28]. Besides this functional information, the radicals also have positional information, that is, the radicals should obey positional regularity indicating the legality of the character [17]. Violation of the positional regularity often provides sufficient information to make the judgment that the character is illegal [29]. Understanding the regularities of these radicals should be very useful for children to access and decode Chinese orthography rapidly and successfully. Shu and Anderson [30] found that children in primary school were aware of the relationship between a semantic radical and the meaning of a character, and were able to use it to correctly derive the meaning of the character that they were unfamiliar with. The relationship between the sound of the phonetic radical and the pronunciation of the whole character which contained the phonetic radical could also be used by 2^nd^ grade children, to obtain the pronunciations of consistent/regular characters [31]. Tong, McBride-Chang, Shu, et al. [32] conducted a longitudinal study of kindergarten children, and found that orthographic awareness skills had consistently made a significant contribution to concurrent and subsequent Chinese character reading development, but that phonological awareness was not significant. However, a similar study conducted by Lin, McBride-Chang, Shu, et al. [33] revealed a different answer to the same question. The regression analysis conducted on their data showed that the syllable deletion task was a significant factor for reading at kindergarten, whereas the orthographic task was strongly associated with reading in primary school. Consequently, Ho et al. [34] suggested that orthographic awareness is one of the four core reading components required in Chinese. Learning to read Chinese characters is not accomplished by the rote memorization of the relationship between print and sound, but rather through understanding the underlying rules of Chinese orthography [35]. It is therefore reasonable to associate orthographic awareness with Chinese reading ability.

### The Present Study

The first aim of the current study was to determine which metalinguistic awareness skills such as orthographic, phonological and morphological awareness exert a unique influence on the acquisition of literacy in Chinese. Do orthographic awareness skills play a more important role than the other two metalinguistic awareness skills, or do morphological awareness or phonological awareness skills exert a stronger influence in reading Chinese? For significant correlations among the three metalinguistic awareness skills, it is important to control any two of them to observe the unique contribution of the third metalinguistic awareness skill. Further, rapid automatized naming, phonological memory and nonverbal intelligence as control variables should also be measured, since they also have been shown as influential predictors in the acquisition of reading skills in Chinese [13], [14], [36].

The second aim was to determine if these metalinguistic awareness skills would exert their influence at different developmental stages in the acquisition of literacy in Chinese. Models of English word-reading development along with empirical evidence supported the idea that the independence and importance of the contribution of phonological, morphological, and orthographic awareness skills to word reading varied with age (e.g., [10], [37], [38]). Ho et al. [17] also put forward a three-stage model of Chinese reading acquisition (similar to the three-stage model of reading development in English postulated by Frith [39]) based on their studies of developmental dyslexia in Chinese. In Ho et al.'s three-stage model of reading development in Chinese, the first logographic stage takes phonological memory and visual processing skills as the important factors, the second cipher stage takes orthographic skill as the important factor, and the third orthographic stage takes rapid naming skills as an important factor. Each stage differs in the way different cognitive processes involved in reading Chinese exert an influence. However, neither the currently available research on English word reading nor studies on Chinese dyslexia are suited to the understanding of the processes involved in the acquisition of reading skills of typically developing Chinese children. Further, given the wide diversity of Chinese learning environments (e.g., teaching instructions) [40], Chinese literacy development between Hong Kong and Mainland China may be different. It is therefore necessary to observe cross-sectional reading development in Chinese children especially in Mainland China from kindergarten to Grade-3 primary school children in order to ascertain the developmental trajectory in the acquisition of literacy in Chinese.

## Methods

### Participants

411 children from two primary schools in Shijiazhuang, the capital of Hebei province participated in the study. 20 children were excluded from the analyses due to their incomplete data. The remaining participants consisted of 101 preschool children (mean age = 72.72 m, SD = 5.28 m), 94 Grade-1 children (mean age = 85.08 m, SD = 6.00 m), 98 Grade-2 children (mean age = 96.00 m, SD = 5.28 m), and 98 Grade-3 children (mean age = 109.32 m, SD = 6.84 m). All children were Mandarin speakers. None of the children were reported to have a history of language or speech or hearing problems by their teachers. Written consent was obtained from the children's parents and their teachers. The research project was approved by the Research Ethics Committee of the Institute of Psychology, Chinese Academy of Sciences.

### Procedures and Measures

The materials consisted of a battery of tests covering phonological awareness, orthographic awareness, morphological awareness, naming, phonological memory and character recognition. Each test is described in detail below. All the tasks were administrated within two sessions, and each session took about 40 minutes.

#### Phonological Awareness Tasks

Oddity Detection task and Deletion task were used to measure (i) tone awareness, (ii) onset-rime awareness and (iii) phonemic awareness. The tasks that measure different levels of phonological awareness were included in order to prevent floor and ceiling effects when the same measures were given across a wide range of children's ages following Gottardo, Yan, Siegel, & Wade-Woolley [41].

In the Oddity Detection test, the children were asked to find the odd syllable within three stimuli presented orally twice by the experimenter. For example, /hao3/ is the odd one in the set /tan2/, /gan4/, /hao3/.There were two practice and 14 experimental trials. This test showed reliability of 0.60 (Cronbach's alpha). In the Deletion test, the children were asked to say a syllable aloud after deleting a specified phoneme, onset or rime of a character (i.e., syllable) presented orally by the experimenter. For example, say /miao2/ without the /i/ sound. Prior to 14 experimental trials, two practice trials were given. The stimulus items were based on the phonological tests by Liu, Liu, & Zhang [42] and Shu et al. [25]. The internal consistency coefficient of this test was 0.86.

#### Morphological Awareness Tasks

Morphological awareness skills were measured by a morphological construction test and a morpheme judgment test. The Chinese morphological construction test was similar to a sentence analogy test (see below), which was often used to measure morphological awareness skills in English [43], [44]. *Sentence A* and *Sentence B* were orally presented to the participants, indicating a certain morphological relationship, such as, “Early in the morning we can see the sun rising. This is called a sunrise.” Then *Sentence C* was presented to create a new scenario, and children were asked to produce a new word or concept to complete *Sentence D* based on the morphological relationship. For example, *Sentence C* and *D* for this trial would be “At night, we might also see the moon rising. What could we call this?” The correct answer is moonrise. There were 15 stimulus items, six of which were adapted from McBride-Chang et al. [21]. The reliability of this test was 0.76 (Cronbach's alpha).

Morpheme judgment test was used to investigate children's ability to understand the different meanings of one character as a constructed morpheme in different words. For example, “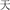/tian1/” is the constructed morpheme for two-character compound words “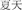/xia4tian1/” (meaning summer) and “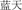/lan2tian1/” (meaning blue sky). The two words sharing the same character were presented to children simultaneously, and the children were asked to judge whether the common character is homo-morpheme or not.

There were two practice trials prior to 30 experimental trials, half of which were from Shu et al. [25], and the other half were from Ku and Anderson [45]. The reliability of the test was 0.87 (Cronbach's alpha).

#### Orthographic Awareness Tests

Similar to the previous studies [17], [41], [46], [47], levels of orthographic awareness skills were measured by the print knowledge test and the lexical decision task. Both tests were two-alternative, forced-choice discrimination tasks where the children were asked to decide which one looked more like a real character between a non-character which violated orthographic regularities and a pseudo-character which was made up based on the rules of Chinese orthography.

Print knowledge of Chinese characters refers to the rudimentary knowledge of Chinese orthographic regularity, that is, understanding of the rudimentary orthographic regularities/conventions of printed Chinese characters. The test consisted of 45 two-alternative stimulus items, in which non-characters violated certain print conventions of Chinese characters (see Table S1 for more details). This test showed a moderately high reliability of 0.79 (Cronbach's alpha).

In the lexical decision task, the children were asked to decide if stimulus characters were pseudo-characters or non-characters. Non-characters were constructed by violating the Chinese orthographic regularities/conventions, while pseudo-characters accorded with the orthographic rules (see Table S2 for more details). The stimuli were adapted from Peng, Li, and Yang [48]. The reliability (Cronbach's alpha) of the task was 0.85.

#### Reading

Character Recognition test were used to measure reading ability. 110 Chinese characters were used as stimuli, 100 of which were selected from *Xiandai hanyu pinlv cidian (1986)* (Contemporary Chinese Frequency Dictionary) by the stratified random sampling method used by Chen [49], and Huang and Hanley [50]. Another ten characters which are easily read by most children in kindergarten were selected from the materials used by Chi [51] in order to prevent the floor effect. The children were asked to read these 110 stimulus words aloud as accurately as possible. The number of correct responses was summed up to represent the children's reading ability. The reliability for this task was high (Cronbach's alpha = 0.99).

In addition, rapid automatized naming, phonological memory and nonverbal intelligence were measured as control variables. Naming performance was measured by a continuous number-naming task similar to that used by Denckla and Rudel [52], [53]. Five Arabic digits (2, 4, 6, 7, and 9) were ranged in random order in a row, and each trial consisted of six rows. The children were given two trials, and asked to name these digits as quickly and accurately as possible starting with the top row and going down from left to right. The time taken for each participant to complete the task was recorded using a stopwatch. Phonological short-term memory was measured by the pseudo-word repetition test, in which the children were asked to repeat what they had just heard from the experimenter. There were 27 pseudo-words constructed by stringing two to ten acceptable Chinese syllables together. A two-syllable pseudo-word was used as a practice trial. If the children failed to repeat three consecutive pseudo-words, the session terminated. The Chinese version of the Combined Raven's Test [54] was used to measure the children's nonverbal intelligence.

## Results

Table 1 shows means (M) and standard deviations (SD) of all the tests. The scores in all cases are raw data. Table 2 shows the correlation coefficients among different measured variables.

**Table 1:** Means and Standard Deviations for All Tasks performed by the Participating Children.

Variable (maximum scores)	Preschool		Grade-1		Grade-2		Grade-3	
	M	SD	M	SD	M	SD	M	SD
Nonverbal intelligence (60)	20.51	7.06	30.39	11.30	37.83	11.58	42.76	11.73
Character recognition (110)	15.29	9.72	38.00	17.85	67.63	14.31	81.99	9.35
Phonological awareness								
Oddity detection (14)	5.86	2.40	6.77	2.57	7.26	2.31	8.43	2.52
Deletion (14)	3.10	3.65	8.38	2.97	9.22	2.62	9.86	2.21
Morphological awareness								
Morphological constructions (15)	6.74	2.91	8.30	2.20	9.81	2.05	10.83	1.82
Morpheme judgments (15)	5.48	4.65	7.87	4.39	10.73	2.80	11.67	2.00
Orthographic awareness								
Print knowledge (45)	39.35	3.92	43.46	1.75	44.34	0.91	44.30	1.00
Lexical decisions (30)	18.85	4.63	23.86	3.72	27.31	1.85	28.38	1.25
Rapid automatized naming	0.81	0.22	0.58	0.13	0.50	0.12	0.42	0.08
Oral pseudo-word repetition (27)	13.04	3.07	13.73	3.18	13.78	3.26	14.95	3.13

*Note.* All the figures are raw data.

*P<.05,

**P<.01,

***p<.001.

**Table 2:** Correlations among Individual Tasks.

Variable	1	2	3	4	5	6	7	8
Phonological awareness								
1. Oddity detection	–							
2. Deletion	0.42**	–						
Morphological awareness								
3. Morphological construction test	0.32**	0.56**	–					
4. Morpheme judgment test	0.23**	0.43**	0.47**	–				
Orthographic processing skills								
5. Print knowledge	0.26**	0.55**	0.45**	0.48**	–			
6. Lexical decision	0.37**	0.63**	0.55**	0.51**	0.74**	–		
7. Rapid automatized naming	−0.31**	−0.57**	−0.46**	−0.47**	−0.59**	−0.65**	–	
8.Oral pseudo-word repetition	0.14**	0.14**	0.25**	0.21**	0.16**	0.18**	−0.12*	–

*P<.05,

**P<.01.

Table 3 shows the correlation coefficients between Character Recognition and all the tasks across four different age groups of children, namely, Preschool/Kindergarten, Grade-1, Grade-2, and Grade-3 children respectively.

**Table 3:** Correlations between Character Recognition and Individual Linguistic Tasks at Each Grade.

Variable	Preschool	Grade-1	Grade-2	Grade-3
Phonological awareness				
Oddity detection	0.29**	0.12	0.42**	0.15
Deletion	0.45**	0.19	0.31**	0.30**
Morphological awareness				
Morphological construction test	0.29**	0.27**	0.32**	0.37**
Morpheme judgment test	0.29**	0.31**	0.33**	0.19
Orthographic awareness				
Print knowledge	0.55**	0.31**	0.24*	0.04
Lexical decision	0.63**	0.48**	0.17	0.15
Rapid automatized naming	−0.56**	−0.26*	−0.39**	−0.21*
Oral pseudo-word repetition	0.05	0.12	0.24*	0.31**

*P<.05,

**P<.01,

***p<.001.

The results revealed the following: for Preschool and First-grade children, character recognition skills were significantly related to Lexical Decisions, r = .63, *p*<.01 for Preschool, and r = .48, *p*<.01 for Grade-1 children, but for Grade-2 children, the skills were significantly related to the Oddity Test, r = .42, *p*<.01, and for Grade-3 children, the character recognition skills were significantly related to the performance in Morphological Construction Test, r = .37, *p*<.01.Thus, a significant and positive relationship between the character recognition skills and orthographic awareness skills was found in younger (Preschool and Grade-1) children, but not in the older (Grade-2 and Grade-3) children. Conversely, a significant positive relationship between the character recognition skills and Pseudo-word Repetition was found in older (Grade-2 and Grade-3) children, r = .24, *p*<.05 for Grade-two, and r = .31, *p*<.01 for Grade-3 children, but not in the younger children.

Table 4, Table 5 and Table 6 show the results of the two-step fixed-order hierarchical multiple regressions in order to estimate the variance of Character Reading Skills accounted for by phonological awareness, orthographic awareness or morphological awareness for different age group children. Table 4 revealed that the Orthographic Awareness contributed 11.7% of the variance of the Character Reading for Preschool children, and 8.2% of the variance for Grade-1 children. Table 5 revealed that Phonological Awareness contributed 4.2% of the variance of the Character Reading for Preschool children, and 6.4% of the variance for Grade-2 children. Table 6 revealed that the only Morphological Awareness contributed 6.8% of the variance of the Character Reading for Grade-3 children.

**Table 4:** Hierarchical Multiple Regressions Analyses that estimated the Predictive Power of Orthographic Awareness at Each Grade on Character Reading after Controlling for Differences in IQ, Phonological Awareness, Morphological Awareness, RAN, and Phonological Memory.

Step	Variable	Dependent measure (ΔR^2^)			
		Preschool	Grade-1	Grade-2	Grade-3
1	Nonverbal intelligence	0.489***	0.306***	0.389***	0.270***
	Oddity detection				
	Deletion				
	Morphological structure test				
	Morpheme judgment test				
	Rapid automatized naming				
	Oral pseudo-word repetition				
2	Print knowledge	0.117***	0.082***	0.018	0.015
	Lexical decision				

Note. At step-one, 7variables were entered in the analysis. They were Nonverbal intelligence, Oddity detection, Deletion, Morphological structure test, Morpheme judgment test, Rapid automatized naming & Oral pseudo-word repetition. At step-two, two orthographic awareness tests were entered into the equation. In this way, it was established whether the orthographic factors have a unique contribution to Chinese reading achievement.

*P<.05,

**P<.01,

***p<.001.

**Table 5:** Hierarchical Multiple Regressions Analyses that estimated the Predictive Power of Phonological Awareness at Each Grade on Character Reading after Controlling for Differences in IQ, Orthographic Awareness, Morphological Awareness, RAN, and Phonological Memory.

Step	Variable	Dependent measure (ΔR^2^)			
		Preschool	Grade-1	Grade-2	Grade-3
1	Nonverbal intelligence	0.564***	0.381***	0.343***	0.265***
	Lexical decision				
	Print knowledge				
	Morphological structure test				
	Morpheme judgment test				
	Rapid automatized naming				
	Oral pseudo-word repetition				
2	Oddity detection	0.042**	0.007	0.064*	0.019
	Deletion				

Note. At step-one, 7variables were entered in the analysis. They were Nonverbal intelligence, Print knowledge, Lexical decision, Morphological structure test, Morpheme judgment test, Rapid automatized naming & Oral pseudo-word repetition. At step-two, two phonological awareness tests were entered into the equation. In this way, it was established whether the orthographic factors have a unique contribution to Chinese reading achievement.

*P<.05,

**P<.01,

***p<.001.

**Table 6:** Hierarchical Multiple Regressions Analyses that estimated the Predictive Power of Morphological Awareness at Each Grade on Character Reading after Controlling for Differences in Age, IQ, Phonological Awareness, Orthographic Awareness, RAN, and Phonological Memory.

Step	Variable	Dependent measure (ΔR^2^)			
		Preschool	Grade-1	Grade-2	Grade-3
1	Nonverbal intelligence	0.601***	0.363***	0.374***	0.217**
	Oddity detection				
	Deletion				
	Print knowledge				
	Lexical decision				
	Rapid automatized naming				
	Oral pseudo-word repetition				
2	Morphological structure test	0.004	0.025	0.032	0.068*
	Morpheme judgment test				

Note. At step-one, 7variables were entered in the analysis. They were Nonverbal intelligence, Oddity detection, Deletion, Print knowledge, Lexical decision, Rapid automatized naming & Oral pseudo-word repetition. At step-two, two morphological awareness tests were entered into the equation. In this way, it was established whether the orthographic factors have a unique contribution to Chinese reading achievement.

*P<.05,

**P<.01,

***p<.001.

Finally, in order to directly compare the relative contributions of Phonological, Morphological, and Orthographic Awareness, hierarchical multiple regressions were conducted with the following variables being controlled: Nonverbal Intelligence, Naming and Phonological Memory. The data from the two Phonological Awareness tests, the two Morphological Awareness tests, and the two Orthographic awareness tests were entered in the equations separately in the final step. The results of the analyses are shown in Table 7.

**Table 7:** Hierarchical Multiple Regressions Analyses that estimated the Predictive Power of Phonological Awareness, Morphological Awareness and Orthographic Awareness at each Grade on Character Reading After Controlling for Differences in IQ, Rapid Automatized Naming, Visual Recognition, and Oral Pseudo-word Repetition.

Step	Variable	Dependent measure (ΔR^2^)			
		Preschool	Grade-1	Grade-2	Grade-3
1	Nonverbal intelligence	0.350***	0.262***	0.253***	0.177**
	Rapid automatized naming				
	Oral pseudo-word repetition				
2	Oddity detection	0.117***	0.020	0.104***	0.026
	Deletion				
2	Morphological structure test	0.047*	0.036	0.060*	0.073*
	Morpheme judgment test				
2	Print knowledge	0.207***	0.086**	0.028	0.014
	Lexical decision				

*Note.* In order to identify the unique contributions of phonological awareness (PA), orthographic awareness (OA), and morphological awareness (MA), at step-one, three variables were entered in the equation in order to control for their variance. At step-two, PA (Oddity detection & Deletion), MA (Morphological structure test &Morpheme judgment test), and OA (Print knowledge & Lexical decision) were entered in the analysis respectively.

*P<.05,

**P<.01,

***p<.001.

For Preschool children, Phonological Awareness, Morphological Awareness and Orthographic awareness accounted for 11.7%, F (2, 95) = 10.44, *p*<.001; 4.7%, F (2, 95) = 3.70, *p*<. 05; and 20.7%, F (2, 95) = 22.21, *p*<.001 of the variance of Character Reading, respectively.

For Grade-1 children, neither Phonological Awareness nor Morphological Awareness accounted for a significant portion of the variance of Character Reading. Orthographic awareness was the only significant predictor for Character Recognition in these children, which accounted for 8.6%, F (2, 88) = 5.80, *p*<.01 of the variance.

For Grade-2 children, two significant predictors were Phonological Awareness and Morphological Awareness, which accounted for 10.4%, F (2, 92) = 7.47, *p*<. 01 and 6.0%, F (2, 92) = 4.00, *p*<.05 of the variance respectively in Character Recognition. For Grade-3 children, Morphological Awareness was the only significant predictor of the variance for Character Recognition, which accounted for 7.3%, F (2, 92) = 4.48, *p*<.05.

## Discussion

The present study revealed that reading development through preschool/kindergarten to third-grade Chinese children was influenced by a comprehensive set of cognitive skills. The relationship between reading ability and these different metalinguistic skills appears to change with age. In the following section each of the important cognitive skills identified in the current study in relation to the literacy acquisition/development in Chinese are discussed in turn.

### The Role of Phonological Awareness in Chinese Character Reading

As with the previous studies (e.g., [12], [14]), the current results also showed that phonological awareness uniquely contributes to Chinese reading acquisition. Further, the role of phonological awareness became more important in the later stages in the development of reading skills in Chinese. This is similar to the finding from a similar cross-sectional study conducted by Uno, Wydell, Haruhara, Kaneko and Shinya [55] in Japanese logographic Kanji (originated from Chinese character). They tested Japanese primary school children from Grade-2 (aged 8) to Grade-6 (aged 12) for their abilities to read/write in syllabic Kana and logographic Kanji, and for other cognitive abilities including arithmetic, visual-spatial and phonological processing skills as well as for their size of vocabulary. Uno et al. pointed out that many studies in English (e.g., [56]–[58]) have shown that better phonological awareness leads to better literacy skills, and this relationship emerged relatively early in English [59]. However this relationship was only seen in the older children in Uno et al.'s study [55] in Japanese Kanji (with Japanese children aged 12) as well as in the current study in Chinese (with Chinese children aged eight). Because each Chinese character has a one-to-one correspondence with a single Chinese syllable, and the syllable is a strongly salient unit in spoken Chinese, children seldom need to analyze the syllable into smaller phonological units (such as phonemes) at their earliest years of literacy acquisition. Consequently, they need more time and practice to develop phonological awareness.

Moreover, the current results showed that phonological awareness significantly predicts the character recognition at preschool and Grade-2, but not at Grade-1 and Grade-3. (Note however the contribution of the orthographic awareness was greater than that of phonological awareness in preschool children.) Similarly, Siok and Fletcher [14] showed the predictive power of phonological awareness at Grade-2 and Grade-5, but not at Grade-1 and Grade-3. The developmental discontinuity (i.e., non-linear relationship between Phonological Awareness and character recognition skills) was also seen in the study conducted by Nagy, Berninger and Abbott [60] investigating the relationship between morphology and reading. They pointed out that this developmental discontinuity is normal since between the initial developmental gains and a growth spurt or mastery, there is ordinarily a period of relatively little growth.

### The Role of Morphological Awareness in Chinese Character Reading

From Table 6 and Table 7, it is evident that the contribution of morphological awareness to character reading increased with age. Especially for the Grade-3 children, the oldest children in this cohort, morphological awareness was the only significant predictor for character reading. Thus, the current study further lends support to the previous findings whereby the Morphological Awareness is one of the most important predictors for reading development/acquisition in Chinese (e.g., [24], [25]). The morphologically fairly transparent semantic system and relatively consistent character-to-morpheme correspondences make morphological awareness particularly important for the acquisition of literacy in Chinese [19]. The children with a better understanding of the construction of Chinese words could learn unfamiliar characters quickly and efficiently. Because morphological awareness develops with reading experience [45], and the contribution of other factors decreased with age, morphological awareness plays an increasingly important role with age in Chinese character learning/reading. A similar trend can be seen in English [61] despite the great differences between these two morphological systems.

### The Role of Orthographic Awareness in Chinese Character Reading

The unique contribution of Orthographic Awareness independent of the other factors was identified in this study. The importance of orthographic processing skills in reading in Chinese has been discussed by other researchers (e.g., [14], [29]). However, there are some major differences between previous studies and the current study. For example, (i) previous studies did not include younger children as participants while the current study did, and therefore the former studies were not able to show a developmental trajectory; and (ii) it was not possible for these previous studies to identify the unique contribution of Orthographic Processing skills to reading in Chinese which is independent of (or over and the above) the other factors.

As shown in Table 4, the results revealed that even after controlling most of the predictive factors and other metalinguistic awareness skills, Orthographic Awareness still made a unique and significant contribution to Chinese reading for younger children. Without regular orthography-phonology correspondence in Chinese, children who start to read in Chinese are forced to resort to learn different characters and their corresponding sounds by rote. As the reading development of these children progresses, the children realize the rote learning strategy is not adequate, as many polyphonic characters exist in Chinese. The children therefore start to develop another strategy which is based on the information provided by the phonetic and semantic radicals, which are orthographically less complex. Although the relationship between pronunciation of a radical and the pronunciation of the whole character is not always congruent, the children who understand the orthographic regularities (i.e., congruencies) will establish the orthographic representations efficiently.

However, the contribution of orthographic knowledge to reading acquisition appeared to become insignificant for older children (i.e., after Grade-2). This developmental pattern in Chinese is very different from that of English. In English, the acquisition of the orthographic knowledge requires sufficient print exposure [62], and its contribution to reading acquisition increases with age [63]–[65], even until Grade 8 [38]. This cross-language discrepancy between Chinese and English might be caused by the differences in lexical structure and rules of lexical construction in these two languages. The high scores for print knowledge and lexical decisions in the current study indicated that children could easily understand Chinese orthographic awareness, even for preschool children. Because the radicals can be easily identified visually, Chinese children do not need a lot of print exposure to become familiar with Chinese character regularity compared with English readers. After children became familiar with the information contained in radicals, and the rules of constructing characters, other metalinguistic skills appeared to play a more important role in the acquisition of reading in Chinese, and the contribution of orthographic awareness appeared to diminish. Another reason might be that children initially and typically process Chinese characters in an analytical way, which relies more on orthographic awareness, and as their reading skills develop, they change their decoding process into holistic ways [66].

Caution is needed in drawing any conclusions about the relationship between orthographic awareness and the reading ability of children older than Grade-1 because of the ceiling effect (i.e., some tests were easy for older children). Despite the fact that Ho and his colleagues [29], [47] used a slightly more complex lexical decision task to measure the orthographic knowledge of eight-year-old children, these children also gained high scores in the lexical decision task (mean percentages are higher than 85%). It is therefore necessary to develop more sensitive tasks to measure the level of the orthographic skills in the older Chinese children, and explore the contribution of orthographic awareness to the reading ability of these children.

### The Developmental Stages of Chinese Character Learning through Preschool to Third Grade

As described earlier, Ho et al. [17] postulated a three-stage model of Chinese reading acquisition following the models of acquisition of reading in English (e.g., [39], [67]). According to the model, the first stage is a logographic stage, the second stage is the analytical “cipher stage”, and the third stage is an orthographic stage. These stages are distinguished from each other by the involvement of different metalinguistic skills. However, the current results do not fit this model easily as the most important predictor in the study was orthographic awareness at the beginning of reading acquisition. At this stage, the children became aware that Chinese characters are made up of radicals, understood the positional and functional regularities of radicals, and used these regularities to link the character with the syllable/pronunciation. At the second stage (e.g., during Grade-2), phonological awareness became the most important predictor. Children may need to develop onset-rime awareness in order to utilize an orthographic analogy for reading or some general phonological awareness to learn script-sound regularity [68]. And at the third stage (e.g. nine-years-old children), morphological awareness became the most potent predictor when children with better morphological skills can acquire new Chinese characters/words.

### Directions for Future Research

One of the most logical directions for future research lies in a longitudinal investigation to consolidate the findings obtained from the present cross-sectional study. Although it is acknowledged that the interpretations offered in this study may still be tentative, they provide a general profile of Chinese reading acquisition in young children. Another line of new research lies in the inclusion of other variables such as size of vocabulary (seen for example in the study of Uno et al. [55] for reading development in Japanese Kanji) and syllable awareness (seen in the study of Shu, Peng, & McBride-Chang [69] in Chinese), in addition to the variables used in the current study. Vocabulary is generally considered as an important predictor in reading acquisition. Many researchers reported that vocabulary was closely associated with phonological awareness [70], morphological awareness [19], [21], and orthographic awareness [71].

## Conclusions

Children rely on phonological, orthographic and morphological awareness when acquiring reading skills. For the Chinese children who participated in the current study, orthographic awareness exerted an influence in the early stages of reading development in particular the Preschool and Grade-1 children. It was found that morphological awareness was also closely related to character reading, but only for the older children in the Grade-3. In addition, the predictor factors relating to reading acquisition change continuously as children develop, and for the children in the current study, this was no exception.

## Supporting Information

Table S1 Nine Categories of Non-characters used in the Print Knowledge Test.
(DOC)

Table S2 Three Types of Non-character in the Lexical Decision Task.
(DOC)
