# Supplementary material for: Developmental Changes in the Role of Different Metalinguistic Awareness Skills in Chinese Reading Acquisition from Preschool to Third Grade
Source: PLoS One. 2014 May 8;9(5):e96240. doi: 10.1371/journal.pone.0096240 (PMC4014499; doi:10.1371/journal.pone.0096240)
Supplement: Table S1 — Nine Categories of Non-characters used in the Print Knowledge Test. (DOC) [file pone.0096240.s001.doc]

These 45 items were divided into nine categories of violated conventions: (i) pictures, (ii) geometric drawing combinations, (iii) upside-down radicals or components, (iv) characters with illegal radicals or components, (v) radical-like combinations, (vi) missing strokes, (vii) missing radicals or components, (viii) scribbles and (ix) random combinations of components.

Table S1: Nine Categories of Non-characters used in the Print Knowledge Test

| Violation type | Incorrect item | Correct item |
| --- | --- | --- |
| (i) Pictures | 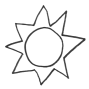 | 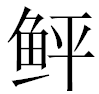 |
| (ii) Geometric drawing combinations | 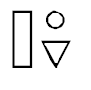 | 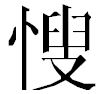 |
| (iii) Upside-down radicals or components | 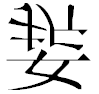 | 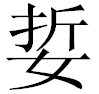 |
| (iv) Characters with illegal radicals or components | 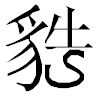 | 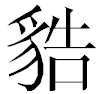 |
| (v) Random combinations of components | 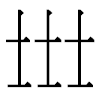 | 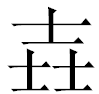 |
| (vi) Radical-like combinations | 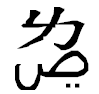 | 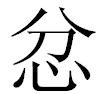 |
| (vii) Scribbles | 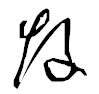 | 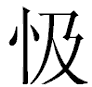 |
| (viii) Stroke missing | 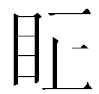 | 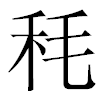 |
| (ix) Radicals or components missing | 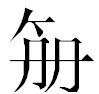 | 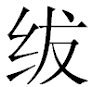 |
