# Supplementary material for: Developmental Changes in the Role of Different Metalinguistic Awareness Skills in Chinese Reading Acquisition from Preschool to Third Grade
Source: PLoS One. 2014 May 8;9(5):e96240. doi: 10.1371/journal.pone.0096240 (PMC4014499; doi:10.1371/journal.pone.0096240)
Supplement: Table S2 — Three Types of Non-character in the Lexical Decision Task. (DOC) [file pone.0096240.s002.doc]

The structure of all the non-characters and pseudo-characters was left-right with two radicals. There were three types of non-characters, each with its radical in a different false position: (i) false right, (ii) false left and (iii) left/right reversal. The number of strokes of the paired items was balanced.

Table S2: Three Types of Non-character in the Lexical Decision Task

| Violation type | Incorrect item | Correct item |
| --- | --- | --- |
| (i) False left position | 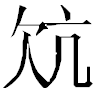 | 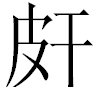 |
| (ii) False right position | 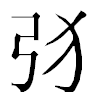 | 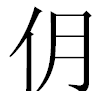 |
| (iii) Left/right reversal | 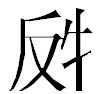 | 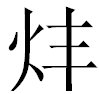 |
